# Polycomb Repressive Complex 2 Regulates MiR-200b in Retinal Endothelial Cells: Potential Relevance in Diabetic Retinopathy

**DOI:** 10.1371/journal.pone.0123987

**Published:** 2015-04-17

**Authors:** Michael Anthony Ruiz, Biao Feng, Subrata Chakrabarti

**Affiliations:** Department of Pathology and Laboratory Medicine, Western University, London, Ontario, Canada; Univeristy of Miami, UNITED STATES

## Abstract

Glucose-induced augmented vascular endothelial growth factor (VEGF) production is a key event in diabetic retinopathy. We have previously demonstrated that downregulation of miR-200b increases VEGF, mediating structural and functional changes in the retina in diabetes. However, mechanisms regulating miR-200b in diabetes are not known. Histone methyltransferase complex, Polycomb Repressive Complex 2 (PRC2), has been shown to repress miRNAs in neoplastic process. We hypothesized that, in diabetes, PRC2 represses miR-200b through its histone H3 lysine-27 trimethylation mark. We show that human retinal microvascular endothelial cells exposed to high levels of glucose regulate miR-200b repression through histone methylation and that inhibition of PRC2 increases miR-200b while reducing VEGF. Furthermore, retinal tissue from animal models of diabetes showed increased expression of major PRC2 components, demonstrating *in vivo* relevance. This research established a repressive relationship between PRC2 and miR-200b, providing evidence of a novel mechanism of miRNA regulation through histone methylation.

## Introduction

Diabetic retinopathy is a microvascular complication of diabetes and a leading cause of vision loss [1]. In early diabetic retinopathy, hyperglycemia induces the expression of vasoactive and inflammatory factors that increase retinal capillary permeability, causing macular edema, and contributing to pericyte and endothelial cell loss [2]. Extracellular matrix (ECM) proteins also are increased, contributing to basement membrane thickening [3]. As diabetic retinopathy progresses, microaneurysms develop and new vessels are formed. New vessels may lead to bleeding and tractional retinal detachment, leading to vision loss. Sustained hyperglycemia, elevated blood pressure and abnormal plasma lipids are all important risk factors in the progression of chronic diabetic complications including retinopathy. However large scale studies in both types of diabetes have shown that hyperglycemia is the main factor in the development of microvascular complications [4–6].

There are numerous signaling events and pathways that are involved in altering the expression of vasoactive factors like vascular endothelial growth factor (VEGF). Glucose, first leads to signaling changes in endothelial cells of the retinal capillaries, which further leads to altered signals to other cell types. Endothelial cells are particularly susceptible to hyperglycemcia-induced damage due to their constitutive expression of glucose transporter 1 (GLUT-1) [7–9]. A number of mechanisms are involved in hyperglycemia-induced damage, including production of advanced glycation end products, oxidative stress and activation of intracellular signaling pathways such as proten kinase C (PKC) and p38 mitogen-activated protein kinase (MAPK) [10–14]. The functional consequence of the altered signaling events in diabetes is increased transcription of multiple vasoactive factors and ECM proteins, which are involved in the development and progression of chronic diabetic complications.

Recent research in our laboratory has elucidated the role of several microRNAs (miRNAs) that are involved in regulating vasoactive factors and ECM proteins in diabetic complications [15,16]. miRNAs are post-transcriptional regulators of gene expression. They are produced initially by RNA Polymerase 2 (Pol2) as immature transcripts and are processed into shorter, mature miRNAs [17,18]. Mature miRNAs target messenger RNAs (mRNAs) for inhibition through specific binding at the 3’ untranslated region (UTR), triggering mRNA degradation or halting translation depending on complementarity [19]. From an evolutionary perspective, miRNAs represent another level of control over cellular events by tightly regulating gene expression [20]. Therefore, aberrant miRNA expression in disease processes can disrupt normal cell physiology and mediate pathogenetic processes [21,22]

One miRNA of importance in diabetic retinopathy is miR-200b. miR-200b has been shown to regulate VEGF [15]. Our laboratory has shown decreased miR-200b in bovine retinal endothelial cells exposed to high levels of glucose, as well as in retinal tissue of streptozotocin-induced (STZ) diabetic rats at 1 month following diabetes induction. Due to loss of negative regulation, VEGF expression and retinal permeability were enhanced. Intravitreal injections of miR-200b were protective by reducing VEGF as well as vessel permeability [15]. Furthermore, miR-200b levels were decreased in human retinal tissue from diabetic patients compared to non-diabetic patients, further supporting that loss of miR-200b occurs in diabetic retinopathy and that restoring miR-200b may be therapeutically useful. However why miRNAs, like miR-200b, become dysregulated in diabetes is poorly understood. Additional research in this topic is necessary to further our understanding and develop novel treatment strategies, and current research suggests an epigenetic link between microRNA regulation and histone methylation.

Histone methylation is another emerging theme in the field of epigenetics and is involved in the coordination of gene expression. Amino acid residues within histone subunit molecules can become methylated by histone methylatransferases, which changes the overall accessibility of chromatin and affects how transcription factors can activate/repress genes in the modified area [23]. So far only H3K4 and H3K9 methylation have been examined in cell culture studies and animal models of diabetes. However, evidence suggests that methylation is indeed a therapeutically targetable mechanism through its involvement in gene regulation in chronic diabetic complications [24–27].

One particular type of methylation, H3K27me3, has been linked to miRNA regulation in several investigations. Polycomb Repressive Complex 2 (PRC2) is a multimeric complex that catalyzes trimethylation of lysine 27 on histone H3 (H3K27me3) and has been linked to negatively regulating many genes, including miRNAs [28,29]. It consists of a major methyltransferase core, EZH2, major adaptor proteins, EED and SUZ12, as well as long-non coding RNA [29–33]. Most of the functional importance of PRC2 has come from research in the cancer field. In a variety of cancers, PRC2 components such as EZH2 and SUZ12 are increased, leading to increased H3K27me3 at a variety of gene promoters. The functional result is silencing of expression in that area, resulting in increased angiogenesis and enhanced metastasis [34]. PRC2 has also been implicated in regulating cell-cell adhesion and inflammatory genes in endothelial cells [35]. Most importantly, PRC2, and specifically the EZH2 and SUZ12 subunits, have been shown to regulate miR-200b in the context of neoplasia [36,37]. Furthermore, in human fibroblasts, increased H3K27me3 was found at the promoter regions of the miR-200 gene cluster with correlating decreased expression relative to epithelial cells, illustrating a mechanism for how miR-200b becomes repressed [38].

While there is limited understanding as to why microRNAs like miR-200b become repressed in diabetic complications, based on the above discussion, it is possible that the underlying regulation of miR-200b may involve PRC2. We hypothesize that glucose induces increased PRC2 expression and activity, which causes alteration of miR-200b levels. Such changes may play a role in the development of diabetic retinopathy. Altogether, the importance of miR-200b in diabetic retinopathy, with possible links of PRC2 to miR-200b, provides clear rationale for investigating this complex in the context of diabetic retinopathy. Involvement of PRC2 in diabetic retinopathy remains elusive, as well as links to VEGF and angiogenesis, providing additional motivation for the current study.

## Materials and Methods

### *In Vitro* Studies

All cell types investigated, including those isolated from diabetic individuals, were obtained from commercial sources. To investigate PRC2 and miR-200b regulation in the context of diabetic retinopathy, the major cell type used for investigation was the human retinal microvascular endothelial cell (HRMECs, Olaf Pharmaceuticals, Worchester, MA, Cat# HEC09). Other endothelial cell types were used for parts of this investigation include human dermal microvascular endothelial cells (HDMECs) isolated from non-diabetic individuals (Lonza, Cat# CC-2543) and type 1 and type 2 diabetic individuals (Lonza, Cat# CC2929, CC2930), were grown and passaged in tissue culture flasks in Endothelial Basal Medium 2 (EBM-2; Lonza, Walkersville, MD) containing 5mM D-glucose, 10% fetal bovine serum (FBS; Sigma-Aldrich, Oakville, ON) and all provided growth factors and antibiotics (EBM-2 Single Quots). No additional antibiotic was added to the medium. HRMECs were incubated at 37°C with 5% CO_2_ and used between passages 3 and 8 to minimize variability. Prior to experimentation, HRMECs were seeded on the appropriate dish based on the assay. At 80–90% confluency, HRMECs were serum starved overnight by removing the growth medium and replacing it with EBM-2 containing 0% FBS, no growth factors or antibiotics. Following starvation, cells were treated with additional D-glucose (Sigma-Aldrich, Oakville, ON) to represent high glucose levels (HG, 25mM) with normal glucose controls (NG, 5mM). Osmotic controls were also used where necessary (20mM L-glucose + 5mM D-glucose). All experiments were performed with 6 replicates unless noted otherwise.

### *In Vivo* Studies

All animal were cared for according to the Guiding Principles in the Care and Use of Animals. All experiments were approved by Western University and Animal Care and Veterinary Services (ACVS). These experiments conform to the Guide for Care and Use of Laboratory Animals published by the NIH (NIH publication no. 85-23, revised in 1996).

Male Sprague–Dawley rats (175 g, 6 weeks old) were obtained (Charles River, Wilmington, MA, USA). Diabetes was induced by a single intraperitoneal injection of streptozotocin (STZ, 65mg/kg, in citrate buffer 5.6pH), with control rats receiving an identical volume of citrate buffer. Rats were monitored for changes in body weight and blood glucose. After 4 weeks of diabetes duration, rats were sacrificed, retinal tissue was collected and RNA was extracted as described below.

Male C57BL/6 mice (23–36gm, were administered three doses STZ (50mg/kg) to induce diabetes, with control animals receiving an identical volume of citrate buffer. Animals were monitored for changes in body weight and blood glucose. After 8 weeks of diabetes duration, mice were sacrificed and retinal tissue was collected.

### RNA Extraction and Real Time RT-PCR

mRNA was extracted using TRIZOL reagent (Invitrogen, Burlington, ON) according to manufacturer’s instructions. RNA concentration was quantified with spectrophotometry (260nm; Gene Quant, Pharmacia Biotech, USA).

To generate cDNA, 2μg of total RNA was used with the High Capacity Reverse Transcription Kit (Applied Biosystems, Foster City, CA) and random hexamer primers. Quantitative Real time RT-PCR was performed using a Roche LightCycler 96 (Roche, Laval, QC) and SYBR Green detection (Clontech, Mountain View, CA). The reaction mixture (total volume 20μL) consisted of the following components: 10μL of SYBR Advantage qPCR Premix, 1μL of each 10μM forward and reverse primer, 1μL cDNA sample and 7μL nuclease free water. Primers for VEGF, EZH2, EED, SUZ12, KDM6A, and KDM6B are shown in Table 1 (Qiagen, Germantown, MD and Sigma-Aldrich). Normalization was performed to β-actin to account for differences in reverse transcription efficiencies of cDNA.

**Table 1:** Oligonucleotide sequences RT-PCR.

Genes	Primer Sequences (5′ → 3′)
β-actin (human/rat/mouse)	CCTCTATGCCAACACAGTGC CATCGTACTCCTGCTTGCTG
VEGF (human)	GAACTTTCTGCTGTCTTGGG CTTCGTGATGATTCTGCCCT
EZH2 (human)	QIAGEN (QT00054614)
EED (human)	QIAGEN (QT00074627)
SUZ12 (human)	TACGGCTCCTATTGCCAAAC TGCTTCAGTTTGTTGCCTTG
KDM6A (human)	QIAGEN (QT00094654)
KDM6B (human)	QIAGEN (QT00098742)
VASH1 (human)	GTTCCCTCCGAAACTGAGAC ACAAAGCACCCCCATCTAAC
EZH2 (rat)	GCACACTGCAGAAAGATCCA AGGTAGCACGGACACTGCTT
EZH2 (mouse)	AGACGTCCAGCTCCTCTGAA CATCCTCAGTGGGAACAGGT
EED (rat/mouse)	CTGGCAAAATGGAGGATGAT GGTCAGTGTTGTGCATTTGG
SUZ12 (rat/mouse)	GTCTCAGGGGTTCCAAAACA ACACTGCCTGTTCCAAATCC

### MiRNA Extraction and Real Time RT-PCR

To isolate miRNA, mirVana microRNA Isolation Kit (Ambion, Austin, TX) was used according to the manufacturer’s instructions. This protocol involves precipitating RNA on a column to reduce the loss of small RNA molecules such as miRNAs. The RNA concentration was quantified using spectrophotometry (260nm).

To generate cDNA, 100ng of total RNA was used with Reverse transcription was performed using the High Capacity Reverse Transcription Kit.

Specific primers are necessary to analyze mature miRNAs due to their short size. TaqMan miRNA Assays (Applied Biosystems) were used as primers for Real Time RT-PCR (Table 2). miR-200b expression was quantified using this method and normalization was performed to house keeping gene U6.

**Table 2:** RT-PCR TaqMan miRNA Probe Sequences.

Genes	Probe Stem-Loop Sequence (5′ → 3′)
mature miR-200b	CCAGCUCGGGCAGCCGUGGCCAUCUUACUGGGCAGCAUUGGAUGGAGUCAGGUCUCUAAUACUGCCUGGUAAUGAUGACGGCGGAGCCCUGCACG
U6	GTGCTCGCTTCGGCAGCACATATACTAAAATTGGAACGATACAGAGAAGATTAGCATGGCCCCTGCGCAAGGATGACACGCAAATTCGTGAAGCGTTCCATATTTT

### Protein Extraction and Western Blotting

To determine changes in VEGF expression at the protein level, Western Blotting was performed. Protein was extracted using whole cell lysates in RIPA buffer, as per manufacturer’s instructions (Sigma-Aldrich). Lysates were sonicated twice for 30 seconds on ice prior to final centrifugation to increase protein yield. Protein concentration was determined using a Bicinchoninic Acid assay (Thermo Fischer Scientific, Rockford, IL).

Western Blotting was performed using 30μg of protein. Samples were diluted LDS Sample Buffer (Life Technologies) and heated at 70°C for 10 minutes. Samples were separated on a 4–12% Tris-Glycine gel (Life Technologies) and were transferred overnight to a PDVF membrane. The membrane was blocked for 30 minutes at room temperature and washed twice in T-PBS (Sigma-Aldrich, 0.1% Tween). Primary antibody incubation (VEGF or β-actin) was performed in blocking buffer at empirically determined dilutions (1:200 and 1:1000 respectively, Santa Cruz Biotechnology, Santa Cruz, CA). Secondary antibody incubation was performed in T-PBS at 1:5000 dilution (Santa Cruz Biotechnology). The membrane was washed four times for 5 minutes after each antibody incubation. Detection was performed using the Amersham ECL Prime Western Blotting Detection Reagent as per manufacturer’s instructions (GE Healthcare Life Sciences, QC, Canada). Luminescence was quantified using densitometry software.

### Chromatin Immunoprecipitation (ChIP) qPCR Analysis

To elucidate changes in chromatin modifications associated with high glucose, ChIP-qPCR was performed. This technique allows pull down of specific protein targets and confirms genome association by performing qPCR with primers against select genomic regions. Chromatin Immunoprecipitation (ChIP) Assay Kit (Milipore, Temecula, CA) was used according to manufacturer’s instructions. Following 24 hours of glucose exposure, the cells were fixed using formalin, collected and lysed in SDS buffer containing protease inhibitors. The lysate was sonicated on ice three times in 30 second intervals with a KONTES Micro-Ultrasonic Cell Disruptor (power of 6 at a tuning of 4) to attain DNA fragments in the 200–1000bp range, appropriate for immunoprecipitation. The lysate was centrifuged and the supernatent was precleared. 5% of each sample was transferred to a fresh tube and stored at -80°C for an input measurement. Antibodies were used in 1:400 dilution for each immunoprecipiation reaction.

Following overnight antibody incubation, Salmon Slurry Agarose was added to the mixture for one hour and spun down. The pellet was washed five times in the following series: Low Salt Solution, High Salt Solution, Li-Cl Solution and twice at room temperature (both 8.0 pH EDTA). Each wash was performed for 5 minutes with rotation. Following the final wash and centrifuge, precipitated DNA was released using of freshly prepared elution solution (1% SDS, 1M NaHCO_3_) with rotation. Cross-links were reversed with high concentrations of NaCl. Proteins were degraded using protease K (Sigma-Aldrich) and DNA was precipitated using phenol-chloroform (1:1). The pellet was resuspended in 8.0 pH EDTA for use in subsequent qPCR analysis.

Pulldown antibodies include anti-H3K27me3 and anti-Pol2 (Milipore). Control for these antibodies include manufacturer provided IgG and ascitic fluid negative control. Primer design to the miR-200b promoter region was performed based on an identified promoter region 2kb upstream of the miR-200b gene (Sigma-Aldrich). Control primers provided by the manufacturer were used to assure the quality of the ChIP reaction, as well as to determine specificity of the chromatin modification changes associated with high glucose. Sequences for these primers are listed in Table 3.

**Table 3:** Oligonucleotides for ChIP-qPCR Analysis.

Genomic region	Primer Sequences (5′ → 3′)
miR-200b promoter	GCCGGGATCACATTCCTC CTCAGCTTGGGAAAATCCAG
Human alpha satellite	CTGCACTACCTGAAGAGGAC GATGGTTCAACACTCTTACA
GAPDH promoter	TACTAGCGGTTTTACGGGCG TCGAACAGGAGGAGCAGAGAGCGA

### VEGF ELISA

To determine changes in secreted VEGF protein, VEGF was measured in the supernatant of HRMECs treated in various conditions. Human Vascular Endothelial Growth Factor ELISA Kit (45-VEGHU-E01, Alpco, Salem, NH) was used according to manufacturer’s instructions. Briefly, this protocol incubates 50μL of supernatant in an antibody-coated well. A biotinylated anti-VEGF antibody was incubated and the wells were washed. Following another series of washes, Streptavidin-HRP solution was incubated in the wells and the wells were washed. Finally, a stabilized chromagen was incubated in the wells in darkness and the reaction was stopped using a stop solution. VEGF concentration was measured with a plate reader at 450nm using a standard curve.

### PRC2 Inhibition with 3-Deazaneplanocin A (DZNep) Chemical Inhibitor

A general methylation inhibitor DZNep (Cayman Chemical, Ann Arbor, MI), which has shown selectivity for PRC2 and H3K27me3, was used as a loss-of-function treatment [32]. DZNep is an indirect inhibitor of methylation, which functions by directly inhibiting a hydrolase that metabolizes Ado-Hcy. Ado-Hcy is a end product formed after a methyltransferase, like EZH2, uses its substrate, Ado-Met, to methylate an amino acid or nucleotide residue [39]. DZNep therefore results in increased cellular levels of Ado-Hcy, which inhibits EZH2 by moving it to an inactive conformation. Concentrations from 1μM to 10μM have been shown to inhibit H3K27me3 activity and 5μM was used for this study [39].

DZNep was tested in normal glucose and high glucose, with an equal amount of DMSO (Santa Cruz Biotechnology) as a control in the same conditions. Cells were pre-treated with DZNep at the starvation period, prior to addition of D-glucose, as well as during the D-glucose treatments.

### PRC2 Gene Knockdown with Small Interfering RNA

To improve the specificity of the hypothesized mechanism, knockdown of specific gene targets was performed using small interfering (siRNA). Specifically, EZH2 and SUZ12 siRNA (Qiagen) were used for gene knockdown with control siRNA (Life Technologies) to control for the transfection process and specificity (Table 4).

**Table 4:** Silencing small interfering RNA sequences.

Gene	siRNA sequences (5′ → 3′)
Control Primers	Life Technologies (AM4635)
EZH2	CCAUGUUUACAACUAUCAATT UUCAUACUUCUAAACAUGGTT
SUZ12	GCAUAAUGUCAAUAGAUAAT UUAUCUAUUGACAUUAUGCTA

Based on previous experiments in our laboratory, cells were treated with siRNA at a concentration of 100nM using Lipofectamine 2000 (Invitrogen) and Opti-MEM (Life Technologies). Cells were grown to 80% confluency and were washed with D-PBS prior to transfection to remove antibiotics. Cells were transfected for 4 hours and was supplemented with 2x the volume of full EBM-2 containing 10% FBS, growth factors and antibiotics. The following day, the cells were serum starved overnight. The following day, cells were treated with D-glucose and were incubated for 48 hours, when they were collected for gene expression analysis as described above. Gene knockdown was verified by RT-PCR.

### Tube Formation Assay

As a measure of VEGF activity and angiogenesis, a tube formation assay was performed with transfected cells. BD Matrigel Matrix Phenol Red-Free (BD Biosciences, Bedford, MA) was aliquoted (100μL per well) into a 96-well plate. Prior to seeding, the transfected cells were collected with trypsin and cellular density was quantified using a hemocytometer. Cells were seeded at a density of 1.5x10^4^ cells per well. Density of cells plated was determined empirically. The cells were then incubated for 1 hour at 37°C to allow for attachment, after which growth medium was aspirated and replaced with serum-free medium containing 25mM (HG) D-glucose. Cells transfected with control siRNA were also cultured in 5mM (NG) D-glucose as a control. After 16 hours incubation, the medium was carefully aspirated from each well, the cells were washed once with D-PBS and pictures were taken at 40× magnification using a Nikon Diaphot microscope (Nikon Canada, Mississauga, ON) with PixeLINK camera and PixeLINK Capture OEM software (PixeLINK, Ottawa, ON). Branch numbers and branch points were counted and a ratio of branches:branch points was calculated for each treatment. Each treatment was repeated in triplicate with at least two field of views per replicate.

### Cell Viability Assay

To determine the cytotoxicity of the treatments used in our experiments, the WST-1 Cell Viability Assay (Roche) was used. Cells were seeded (5.0x10^4^/well) onto a 96-well plate and were allowed to attach overnight. The following morning, the full medium was removed and was replaced with 100μL of serum-free medium containing various concentrations of D-glucose (5mM, 10mM, 15mM, 25mM, 50mM, 100mM). Following 24 hours of incubation with glucose, 10μL of WST-1 reagent was added to each well. The plate incubated for 1.5 hours at 37°C to produce a colour reaction. Absorbance was measured with a Multiskan FC Microplate Photometer (Thermo Scientific, Finland) at 450nm with a reference wavelength of 690nm. Survival was determined by the difference between the absorbances at 450nm and 690nm.

### Statistical Analysis

To determine statistical significance, 2-tailed Student’s T-test was performed with an α-value of 0.05 using SPSS. P values < 0.05 were considered stastically significant. For experiments with multiple comparisons, one-way ANOVA was performed with an α-value of 0.05 using SPSS. Tukey’s test was performed to determine significant differences between groups.

## Results

### MiR-200b and VEGF Are Altered in Human Retinal Endothelial Cells Exposed to High Levels of Glucose

**Fig 1:**
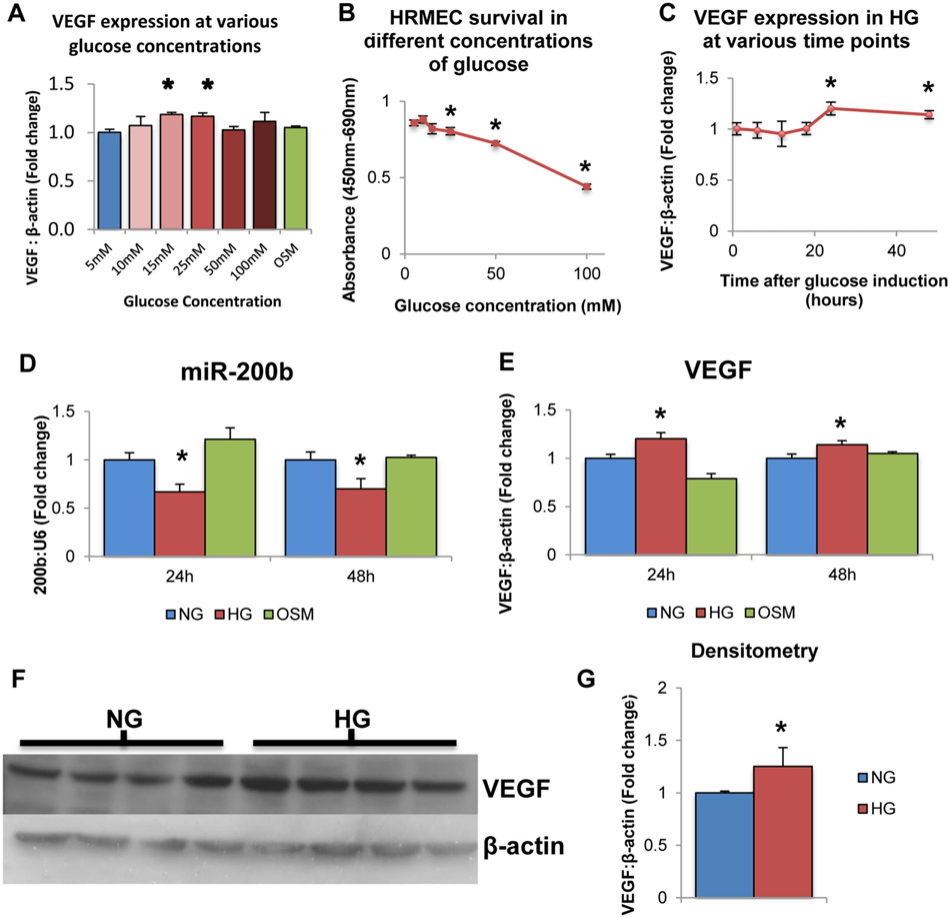
High levels of glucose alter VEGF and miR-200b expression in HRMECs. **A:** HRMECs exposed to various concentrations of D-glucose for 24 hours exhibited differential mRNA levels of VEGF. Compared to 5mM D-glucose, VEGF expression was significantly increased at 15mM and 25mM D-glucose concentrations, with no change at 20mM L-glucose. **B:** Measured by WST-1 assay, HRMECs exposed to increasing concentrations of D-glucose for 24 hours exhibited decreased cell viability at 25mM, 50mM and 100mM compared to 5mM. **C:** HRMECs exposed to 25mM (high glucose; HG) glucose for 24 and 48 hours demonstrated significantly increased VEGF mRNA compared to 5mM (normal glucose; NG). These differences were not observed at time points earlier than 24 hours. **D,E:** HRMECs exposed to 5mM D-glucose (NG) 25mM D-glucose (HG) and 20mM L-glucose+5mM D-glucose (osmotic control; OSM). HRMECs cultured for 24 hours and 48 hours in HG showed significantly decreased levels of miR-200b with parallel increased levels of VEGF expression compared to NG and OSM. **F,G:** VEGF is also increased at the protein level in HG compared to NG as measured by Western Blotting. [* p < 0.05 compared to NG; n = 6; data expressed as mean ± SEM, normalized to β-actin or U6 and expressed as a fold change of NG].

This was one of the first investigations to expose the human retinal microvascular endothelial cell (HRMEC) type to high glucose. Therefore, initial experiments were performed to confirm whether glucose increases VEGF levels. L-glucose treatment (osmotic control; OSM) was also used. L-glucose is metabolically inactive and does not cause oxidative stress and hyperglycemia-induced signaling changes as D-glucose does [15,16,40]. We show that VEGF mRNA levels were significantly increased after 24 hour exposure to medium containing 25mM glucose compared to 5mM glucose, while concentrations greater than 25mM did not show any differences in VEGF expression levels (Fig 1A). 20mM L-glucose did not have any effect on VEGF expression (Fig 1A). Furthermore, increasing glucose cocnentrations showed increased cytotoxicity as measured by a WST-1 assay (Fig 1B). Therefore, 5mM glucose was selected as normal glucose (NG) and 25mM glucose was selected as the high glucose (HG) for all subsequent analyses. To test the effects of glucose at different time points, HRMECs were exposed to 5mM or 25mM D-glucose for various durations. VEGF transcript levels were significantly increased in HG compared to NG at 24 hour and 48 hours time points, though no significant changes were observed before these time points (Fig 1C). Therefore, subsequent experiments were performed at these time points to test the effects of glucose exposure on endothelial cells. These findings are consistent with previous studies performed in our laboratory using various endothelial cell types.

To verify the effects of glucose exposure on miR-200b expression, HRMECs were exposed to 25mM D-glucose for 24 and 48 hours. At both 24 and 48 hours, miR-200b levels showed significant decrease in HG compared to NG and OSM controls (Fig 1D). In parallel, VEGF showed significantly increased levels in HG compared to NG and OSM controls (Fig 1E). This result is consistent with previous work performed in our laboratory in bovine retinal endothelial cells. This is the first time miR-200b decrease has been observed in human endothelial cells isolated from the retina. Furthermore, increased VEGF expression was observed at the protein level measured by Western Blotting (Fig 1F and 1G). This increase at the protein level parallels the increase observed in VEGF mRNA. These findings further strengthen the relationship between hyperglycemia and decreased miR-200b expression in the context of diabetic retinopathy.

### PRC2 Components Are Altered in Endothelial Cells Exposed to High Glucose Levels

Once decreased miR-200b and increased VEGF was established in HRMECs exposed to HG, the mRNA levels of several genes associated with the PRC2 complex was measured by qPCR. These included EZH2, the methyltransferase portion of PRC2, EED and SUZ12, the adaptor protein of PRC2, and KDM6A and KDM6B, which are the demethylases associated with H3K27me3 and thus oppose PRC2 activity.

**Fig 2:**
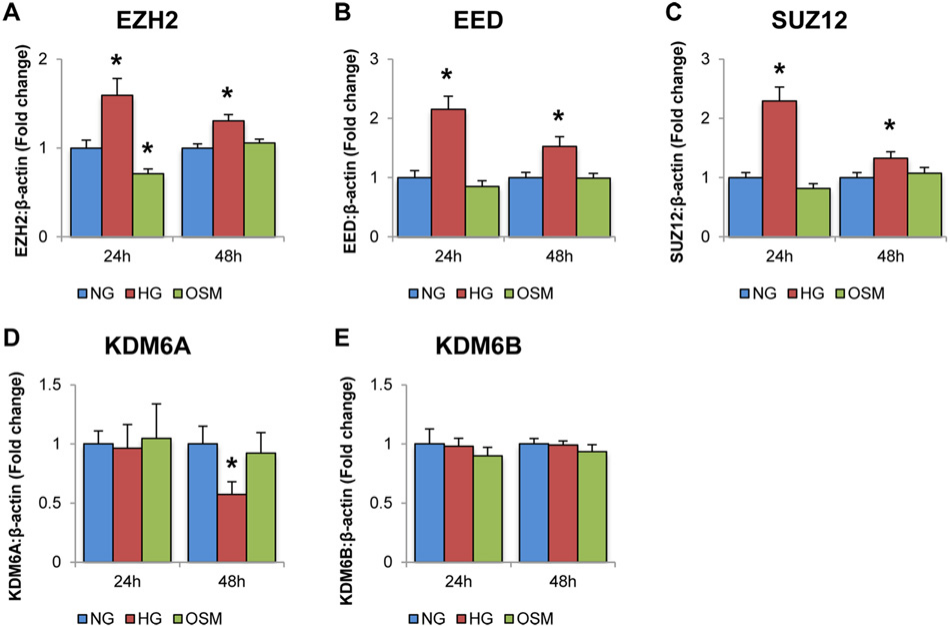
High levels of glucose alter the expressions of PRC2 component and related genes. Real time RT-PCR analysis of PRC2 components in HRMECs exposed to 5mM D-glucose (NG), 25mM D-glucose (HG), and 20mM L-glucose+5mM D-glucose (OSM). **A:** After 24 and 48 hour exposure to D-glucose, levels of EZH2, EED and SUZ12, were significantly increased compared to NG and OSM controls. **B:** After 24 exposure to D-glucose, KDM6A and KDM6B levels did not change in HG. After 48 hours, KDM6A was significantly decreased compared to NG and OSM controls, while KDM6B showed no change. [* p < 0.05 compared to NG; n = 6; data expressed as mean ± SEM, normalized to β-actin and expressed as a fold change of NG].

At both 24 and 48 hours in HG, mRNA of EZH2, EED and SUZ12 were elevated compared to NG and OSM controls (Fig 2A, 2B and 2C). These observations show increased expression of the PRC2 components, which were hypothesized to negatively regulate miR-200b expression. Furthermore, no difference was observed in expression levels of KDM6A and KDM6B between NG, HG and OSM controls (Fig 2D and 2E). Interestingly, KDM6A was observed to significantly decrease in HG at the 48 hour time point (Fig 2D). This suggests there is no change or even a decrease in demethylase expression for H3K27 methylation. Altogether, an increase in expression of PRC2 with no change or decrease in demethylase expression suggests a shift towards increase of H3K27me3 in HG. Overall, these experiments provide correlational evidence linking PRC2 to negatively regulating miR-200b in HG, though additional supporting evidence is necessary.

### PRC2 Activity Is Increased Specifically at the MiR-200b Promoter Region

To demonstrate the involvement of PRC2 at the level of the genome and chromatin modifications, ChIP was performed using antibodies for H3K27me3. Negative control antibody IgG was used to demonstrate specificity of the immunoprecipitation reaction. RNA Polymerase 2 (Pol2) was also immunoprecipitated to measure transcriptional activity of miR-200b at the level of the genome in addition to the RT-PCR results demonstrated above. Ascitic fluid was used as a negative control for this antibody as provided by the manufacturer.

**Fig 3:**
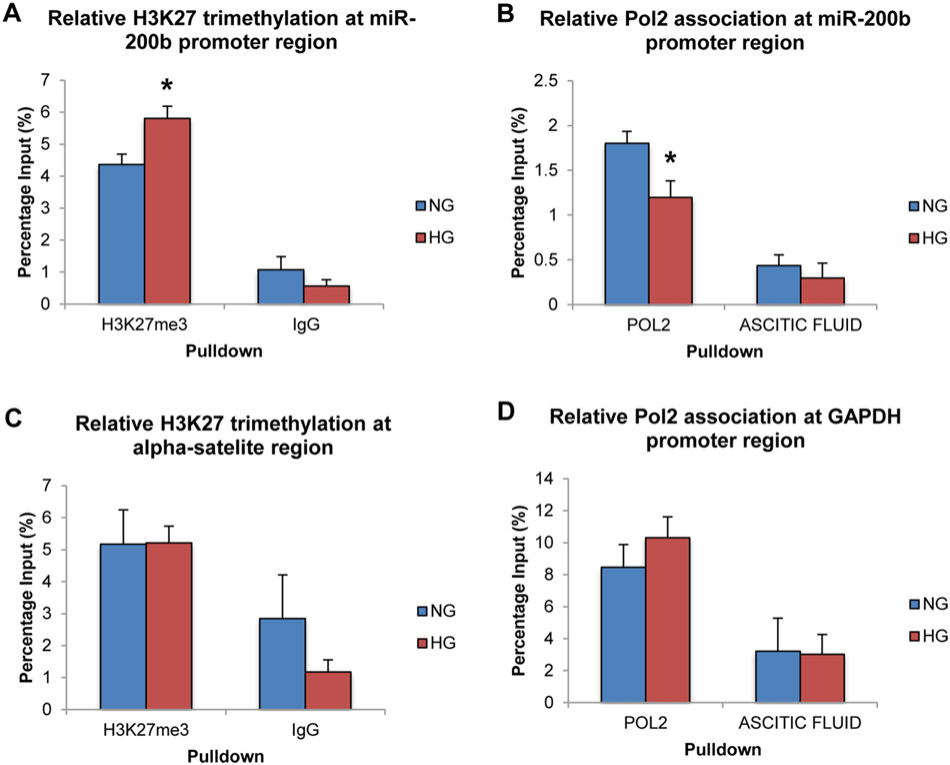
High levels of glucose alter H3K27me3 and RNA polymerase 2 association at the miR-200b promoter region. **A:** ChIP-qPCR analysis of H3K27 trimethylation (H3K27me3) at the miR-200b promoter region. Association of H3K27me3 at the miR-200b promoter region was significantly increased in 25mM (HG) glucose compared to 5mM (NG) glucose. **B:** ChIP-qPCR analysis of RNA polymerase 2 (Pol2) at the miR-200b promoter region. Association of Pol2 at the miR-200b promoter region was significantly decreased in HG compared to 5mM NG. **C:** ChIP-qPCR analysis of H3K27me3 at the human alpha-satelite region. Association of H3K27me3 at the human alpha satellite region showed no significant change between HG and NG. **D:** ChIP-qPCR analysis of Pol2 at the GAPDH promoter region. Association of Pol2 at the GAPDH promoter region showed no significant difference between 25mM (HG) glucose and 5mM (NG) glucose. Association of IgG and ascitic fluid (negative control) was minimal and showed no difference between NG and HG in all experiments. [* p < 0.05 compared to NG; n = 3; data expressed as mean percentage of input ± SEM].

Following ChIP-qPCR analysis, H3K27me3 was found to be significantly increased at the miR-200b promoter region in HG compared to NG controls (Fig 3A). IgG isotype control pull down showed significantly less association than the H3K27me3-specific antibody. Also, Pol2 was found to be significantly decreased at the miR-200b promoter region in HG compared to NG controls (Fig 3B). Again, specificity of this antibody was demonstrated as association with the negative control was minimal. Therefore, this increase of the H3K27me3, which is specific to the PRC2 complex, demonstrates a strong regulatory relationship between PRC2 and miR-200b at the level of chromatin modification. Furthermore, since this type of methylation is believed to be repressing and is associated with closed chromatin, the observation of decreased Pol2 association at the miR-200b promoter region further supports the mechanism and accounts for the decrease in miR-200b expression demonstrated earlier. Altogether, these results paint a picture of the genomic events occurring at the miR-200b promoter region in HG, and further support that PRC2 may regulate miR-200b in diabetes.

Finally, to improve the specificity of this mechanism, areas of the genome with well-known association to the targets that were immunoprecipitated were measured by qPCR. No significant differences were observed in H3K27me3 and Pol2 association at the α-satellite region and GAPDH promoter region between NG and HG, though specificity between the specific and non-specific antibodies was still observed (Fig 3C and 3D). These negative control experiments further support that PRC2 specifically regulates miR-200b in HG, as these changes in H3K27me3 and Pol2 association are specific to certain genomic regions and are not global.

### Loss of Function Analysis Using DZNep Inhibitor Demonstrates a Cause-And-Effect Relationship between PRC2 and MiR-200b Expression

**Fig 4:**
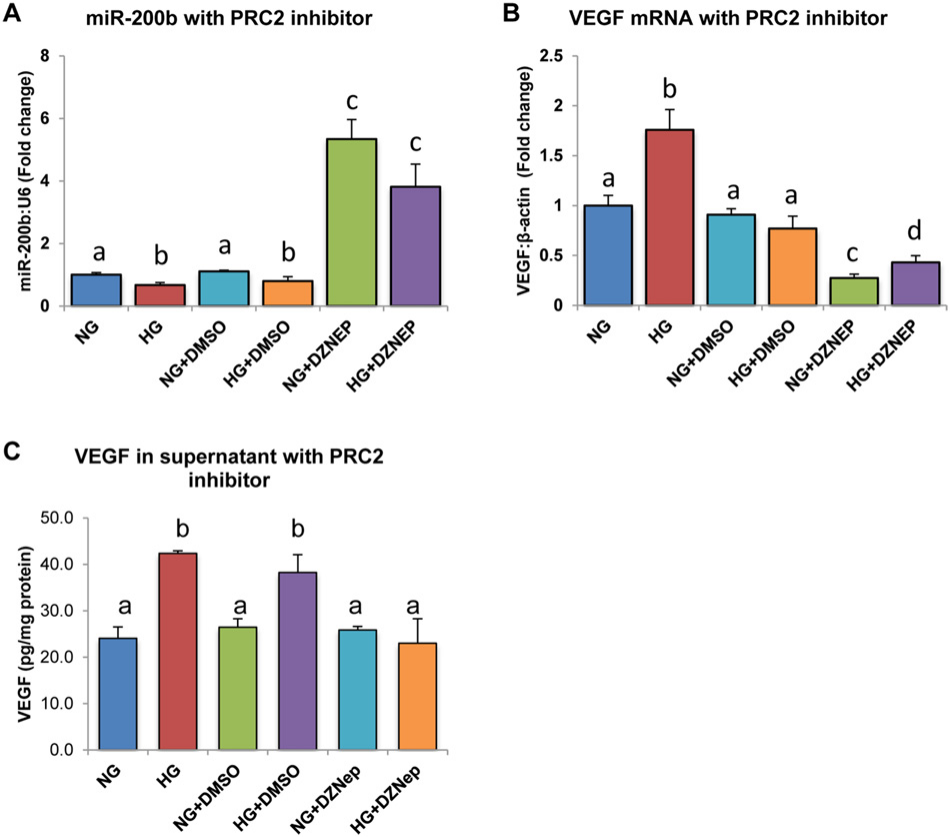
HRMECs treated with 3-Deazaneplanocin A (DZNep) chemical inhibitor demonstrated increased miR-200b and decreased VEGF expression. **A:** After 24 exposure to DZNep in HG, miR-200b levels were significantly increased. **B,C:** In parallel levels of VEGF mRNA and secreted VEGF protein were decreased in HRMECs treated in HG with DZNep compared to controls. [NG = 5mM D-glucose, HG = 25mM D-glucose, NG+DMSO = 5mM D-glucose + 0.05% DMSO, HG+DMSO = 25mM D-glucose + 0.05% DMSO, NG+DZNEP = 5mM D-glucose + 5μM DZNep, HG+DZNEP = 25mM + 5μM DZNep; identical letters represent groups that are not significantly different; p < 0.05; n = 6; data expressed as mean ± SEM, normalized to β-actin for VEGF and U6 for miR-200b, expressed as a fold change of NG].

To demonstrate a cause-and-effect relationship between PRC2 and miR-200b, a chemical inhibitor for H3K27me3, DZNep, was used. HRMECs treated for 24 hours with DZNep showed significantly higher miR-200b levels in both NG and HG compared to the other controls (Fig 4A). In parallel, VEGF mRNA was significantly decreased when HRMECs were treated with DZNep compared to all other controls (Fig 4B). Furthermore, VEGF was measured in the supernatant of treated HRMECs (Fig 4C). VEGF protein was elevated in HRMECs treated in HG and HG with DMSO, compared to respective controls, however this elevation was not seen in HRMECs treated with DZNep, demonstrating parallel changes in VEGF mRNA and protein. Since PRC2 was hypothesized to negatively inhibit miR-200b, through its repressing methylation-mark, increased expression of miR-200b would be expected by inhibiting PRC2. Increased miR-200b expression was observed with decreased VEGF mRNA and protein, a target of miR-200b, providing evidence to support the hypothesis by showing a cause-and-effect relationship by using loss-of-inhibition.

### Loss of Function with SiRNA Demonstrates That SUZ12 Is of Importance in PRC2-Mediated Regulation of miR-200b

**Fig 5:**
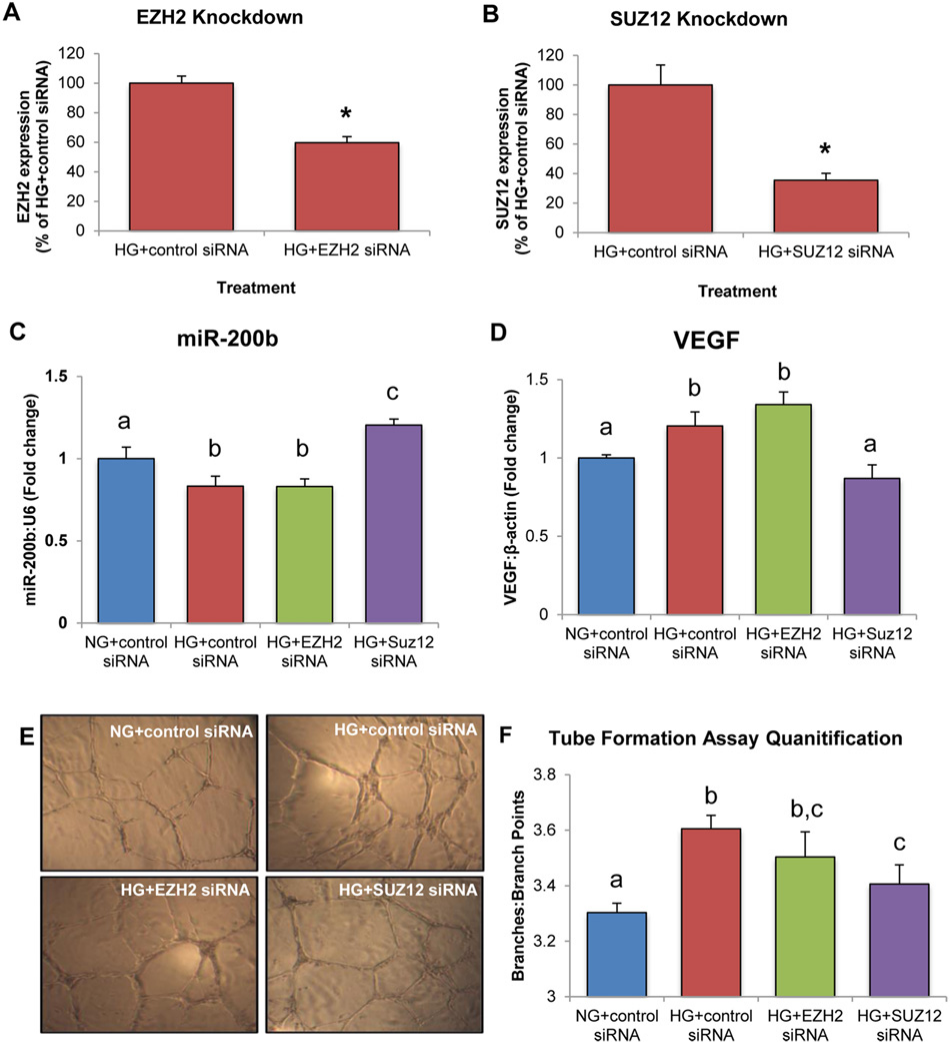
HRMECs treated with SUZ12 siRNA demonstrate increased miR-200b expression, decreased VEGF expression and decreased endothelial branching. **A,B:** Gene knockdown of EZH2 and SUZ12 was confirmed by qPCR. **C,D:** In HRMECs transfected with EZH2 siRNA in HG, miR-200b and VEGF were not significantly different from HG+control siRNA but decreased compared to NG+control siRNA. In HRMECs transfected with SUZ12 siRNA in HG, miR-200b was significantly increased with decreased levels of VEGF compared to HRMECs transfected with control siRNA, with levels similar to NG+control siRNA. **E,F:** Tube formation assay to measure endothelial branching. HRMECs transfected with HG+control siRNA demonstrated significantly increased branching compared to NG+control siRNA. Transfection of EZH2 siRNA did not reduce endothelial branching significantly compared to HG+control siRNA. However, transfection of SUZ12 siRNA significantly reduced endothelial branching compared to HG+control siRNA. [NG+control siRNA = 5mM D-glucose + 100nM control siRNA, HG+control siRNA = 25mM D-glucose + 100nM control siRNA, HG+EZH2 siRNA = 25mM D-glucose + 100nM EZH2 siRNA, HG+SUZ12 siRNA = 25mM + 100nM SUZ12 siRNA; identical letters represent groups that are not significantly different; p < 0.05; n = 6; data expressed as mean ± SEM, normalized to U6 or β-actin and expressed as a fold change of NG+control siRNA].

To further eludicate the role of specific components of PRC2 in the regulation of miR-200b, siRNA-mediated gene knockdown of EZH2 and SUZ12 was performed. Knockdown efficiency was verified by RT-PCR (Fig 5A and 5B). In HG, HRMECs transfected with control siRNA showed a significant decrease in miR-200b expression and increase in VEGF expression (Fig 5C and 5D). When EZH2 was silenced in HG, miR-200b and VEGF showed no differences in expression compared to HRMECs treated with control siRNA in HG (Fig 5C and 5D). However, silencing of SUZ12 increased miR-200b and decreased VEGF, with levels similar to HRMECs transfected with control siRNA in NG (Fig 5C and 5D). In addition, a tube formation assay was conducted to provide a functional correlate. HRMECs transfected with control siRNA and EZH2 siRNA in HG showed significantly increased branching compared to HRMECs transfected with control siRNA in NG (Fig 5E and 5F). HRMECs treated with SUZ12 siRNA in HG showed decreased branching, equivalent to HRMECs treated with control siRNA in NG (Fig 5E and 5F). Altogether, this data suggests that SUZ12 is important in regulating miR-200b in HG, as knockdown of SUZ12 corrected miR-200b and VEGF levels.

### PRC2 Is Altered in Other Endothelial Cell Types and in Target Tissues of Diabetic Complications

**Fig 6:**
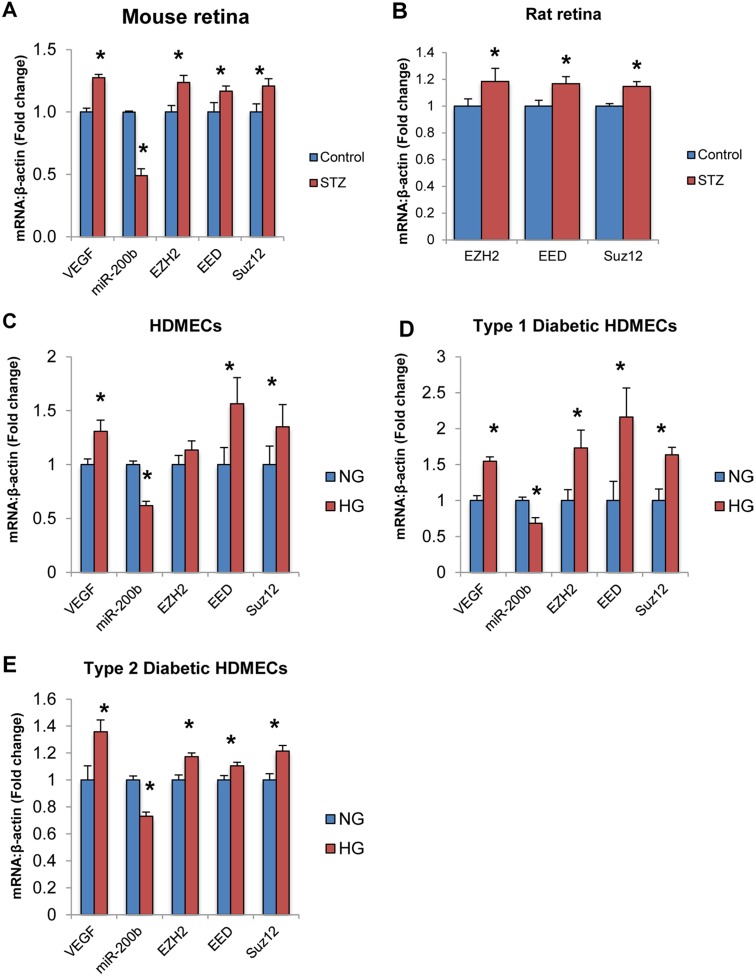
PRC2 components are altered in animal models of diabetes and other endothelial cell types exposed to high glucose. **A,B:** Real time RT-PCR analysis of PRC2 component expression in animal retinal tissue from streptozotocin (STZ) induced diabetic and control animals. **A:** After 1 month of diabetes, VEGF expression was increased and miR-200b expression was decreased. Furthermore, EZH2, EED and SUZ12 levels were increased in rat retinal tissue from diabetic animals compared to control animals. **B:** After 2 months of diabetes, EZH2, EED and SUZ12 levels were increased in mouse retinal tissue of diabetic animals compared to controls. **C,D,E:** Real time RT-PCR analysis of VEGF, miR-200b and PRC2 components in HDMECs of various origins. In all groups, VEGF and miR-200b expression was altered. **C:** In non-diabetic adult HDMECs, EED and SUZ12 levels were significantly increased by HG. **D,E:** In HDMECs isolated from patients with diabetes (Type 1 & Type 2), EZH2, EED and SUZ12 were significantly increased in HG compared to NG controls. [HDMECs = human dermal microvascular endothelial cells, Type 1 = HDMECs isolated from a patient with type 1 diabetes, Type 2 = HDMECs isolated from a patient with type 2 diabetes, * p < 0.05 compared to control/NG; n = 6; data expressed as mean ± SEM, normalized to β-actin and expressed as a fold change of control].

Finally, the expression of PRC2 components was measured in retinal tissues of animal models of diabetes. VEGF expression was found to be increased while miR-200b expression was found decreased in the retinas of diabetic mice compared to controls (Fig 6A). We have previously demonstrated that VEGF and miR-200b expression is altered in the retinas of diabetic rats (21). Furthermore, EZH2, EED and SUZ12 were significantly increased in the retinal tissue of diabetic animals compared to non-diabetic controls (Fig 6A and 6B). These findings are consistent with the *in vitro* data produced in this project and suggests that PRC2-mediated regulation of miR-200b may be relevant *in vivo*.

Furthermore, the expression of PRC2 components was measured in human dermal microvascular endothelial cells isolated from healthy individuals (HDMECs), from a patient with type 1 diabetes (Type 1) and a patient with type 2 diabetes, all treated in NG or HG. These cells show similar alterations in VEGF and miR-200b as in HRMECs (Fig 6C,6D and 6E). Furthermore, significantly increased expression of PRC2 was observed in all cell types treated in HG (Fig 6C,6D and 6E). This suggests that PRC2 may be relevant in endothelial cells and may not be specific to retinal endothelial cells. This opens up the exciting possibility of common pathogenic mechanisms in diabetic complications.

## Discussion

Our key findings are: 1) miR-200b and VEGF are altered in HRMECs exposed to glucose, 2) PRC2 component mRNA and activity at the miR-200b promoter region is in HRMECs exposed to glucose, 3) treatment with DZNep inhibitor and transfection of SUZ12 siRNA increased miR-200b and decreased VEGF levels, and 4) PRC2 components are altered in retinal tissues from diabetic animals and other endothelial cell types exposed to high glucose. Histone methylation as a mechanism for a dynamic response to external stimuli, like hyperglycemia, is a new way of thinking. Few mediators and types of methylation have been studied in the context of diabetic complications thus far. H3K27 methylation research has been expanding in the cancer field but remains uninvestigated in diabetes. Furthermore, recent evidence from cancer research suggests that the major methyltransferase complex for H3K27 trimethylation, PRC2, is involved in silencing several miRNA involved in cancer, including miR-200b. Since miR-200b is also involved in diabetic retinopathy, a similar underlying mechanism may be present and is worth investigating.

Establishment of correlational evidence between miR-200b repression and PRC2 was important to first investigating a potential regulatory relationship. Though many proteins and lncRNA are involved in the PRC2 complex, three major components were selected for investigation. EZH2 is the major methyltransferase component while EED and SUZ12 are the major adaptor proteins. Expression of the major PRC2 components was increased in HG. Furthermore, this increase was specific to D-glucose treatment but not L-glucose. This suggests that PRC2 expression is increased due to signaling induced by hyperglycemia, and not due to osmolarity changes. This could be due to induction of certain transcription factors. For example, hypoxia inducible factor 1 alpha (HIF-1α) has been shown to become stabilized and increase in transcriptional activity by high glucose in brain endothelial cells [41]. Furthermore, HIF-1α has been shown to regulate EZH2 and may be responsible for regulating others genes in the PRC2 complex [42,43]. Further investigation into HIF-1α stabilization would be suggest as a possible upstream mechanism of PRC2 induction in hyperglycemia.

Interestingly, after 24 hours of glucose exposure, no change was observed in the demethylases for H3K27me3, KDM6A and KDM6B. Furthermore, after 48 hours of glucose treatment, KDM6A was decreased relative to NG controls, while KDM6B again showed no change. Although increased mRNA expression of PRC2 components, and no change or decrease in KDM6A/B mRNA, does not imply a direct increase in their activity, it does suggest that PRC2 activity may be increased through increased expression. Further investigating these components at the protein level would strengthen this relationship between high glucose and increased PRC2 expression.

Most importantly, H3K27me3 was increased in HG specifically at the miR-200b promoter region, just upstream of the transcriptional start site. This increase in H3K27 methylation, a repressing type of methylation, suggests formation of closed, inaccessible chromatin and accounts for the decreased expression of miR-200b. Since PRC2 mediates this type of methylation, this observation further strengthens the hypothesis that PRC2 regulates miR-200b in response to hyperglycemia. Furthermore, RNA Pol2 association at the same promoter region was decreased in HG, further suggesting that heterochromatin was formed and that decreased expression of miR-200b in HG is regulated at the genome level. Finally, these changes in qPCR appear to be specific to the miR-200b promoter region. At the human alpha-satelite region, an area of heterochromain well known to associate with H3K27me3, no changes were observed in H3K27me3. Also, at the GAPDH promoter region, a highly expressed area of the genome, no changes were observed in Pol2 association. Therefore, H3K27me3 is increased specifically, while Pol2 association is decreased specifically, at the miR-200b promoter region. This is one of the first investigations to connect two epigenetic mechanism, ie. histone methylation and microRNAs, in the context of a complication of diabetes. It is possible that PRC2 regulates other miRNAs. Other investigations have shown connections to PRC2 and microRNA regulation albeit in a different context [39,44]. In particular, miR-146a, which has demonstrated importance in diabetic retinopathy for its regulation on fibronectin, has been identified as a potential target of PRC2 [15,44]. Thus, it is possible that PRC2 regulates multiple miRNAs. miR-200b is just one miRNA which shows particular relevance in the context of diabetic retinopathy.

Cause-and-effect experimentation was important to further demonstrating a regulatory relationship between PRC2 and miR-200b. Using a chemical inhibitor of PRC2 and H3K27me3, DZNep, miR-200b expression in HG was significantly increased while VEGF mRNA and protein were decreased in parallel. DZNep is not specific for H3K27me3 inhibition, thus siRNA-mediated gene knockdown was used to further elucidate the mechanism [45]. siRNA against EZH2 and SUZ12 were used because these targets have been directly linked to regulating miR-200b in neoplasia. Interestingly, silencing of EZH2 in HG showed no significant changes in miR-200b and VEGF levels when compared to control siRNA. However, in HG, silencing of SUZ12 produced significantly increased miR-200b and decreased VEGF expression compared to control siRNA, and similar expression to control siRNA in NG. Furthermore, silencing of SUZ12 produced decreased endothelial branching compared to control siRNA and EZH2 siRNA, indicating functional alterations in endothelial cells and possibly a proxy of VEGF action. Therefore, it is possible that PRC2 regulation is dependent on SUZ12 and not EZH2. This is interesting because EZH2 is the major methyltransferase component for PRC2 and has been demonstrated to regulate miR-200b in neoplasia. EZH2 is often regarded as the most important component for PRC2 regulation, however in our study SUZ12 was shown to be important for miR-200b regulation. One possibility is that silencing of EZH2 in HG is insufficient to restore miR-200b levels to normal levels because SUZ12 is still able to occupy the miR-200b promoter region. Studies have shown that recruitment of PRC2 is dependent on SUZ12 [33,46,47]. Furthermore, EZH1, a homolog of EZH2, also catalyzes for H3K27me3 and has been shown to compensate for EZH2-knockdown by replacing it in PRC2 complex [40,48]. Thus, silencing of SUZ12 prevents methylation at the miR-200b promoter region by preventing overall recruitment of PRC2, while silencing of EZH2 may be compensated by EZH1. Although EZH1 was not examined in this investigation, it may explain why SUZ12 appears to be important in PRC2-mediated regulation of miR-200b. Additional experimentation is necessary to elucidate the interplay of these specific components of PRC2.

Finally, additional correlational evidence using other models, including animal models and other endothelial cell types, suggests the potential importance of PRC2 in diabetic retinopathy. In retinal tissue from diabetic rats and mice EZH2, EED and SUZ12 expressions were increased relative to non-diabetic control animals. Therefore, this mechanism may be relevant *in vivo* and requires further investigation to determine the therapeutic potential of inhibiting these targets to prevent the microvascular changes associated with diabetes. Furthermore, PRC2 components were elevated in HDMECs exposed to HG isolated from healthy individuals, as well as individuals with type 1 and type 2 diabetes. This further strengthens the mechanism that PRC2 components are elevated in HG in endothelial cells. Furthermore, the increase in HG observed in endothelial cells isolated from diabetic patients offers some interesting conclusions. These cells were isolated from patients who had diabetes for several years (ie. greater than 20 years). However, when treated in HG, PRC2 components were still overexpressed suggesting that hyperglycemia-induced upregulation does not become dampened. Thus, PRC2 and miR-200b may be important in other complications of diabetes.

While other work in the field of epigenetics in diabetic complications has focused on either histone methylation or miRNAs, this project has shown how these epigenetic mechanisms work together to regulate VEGF. This project has demonstrated that PRC2 negatively regulates miR-200b and thus appears to be a good therapeutic target in diabetic retinopathy. Due to its nature as a multimeric enzyme complex, it can potentially be targeted with a small molecule. While much work is needed to design a chemical inhibitor that would selectively target this complex and be effective in being transported to the retina, an organ with its own selective permeability, this work has demonstrated another therapeutic strategy, which may show efficacy in the prevention of diabetic retinopathy. It is hoped that continued investigation into this complex will lead to a small molecule therapy for diabetic retinopathy that can be more accessible and cost effective in treating diabetic retinopathy, though such goals are far in the distance.

**Fig 7:**
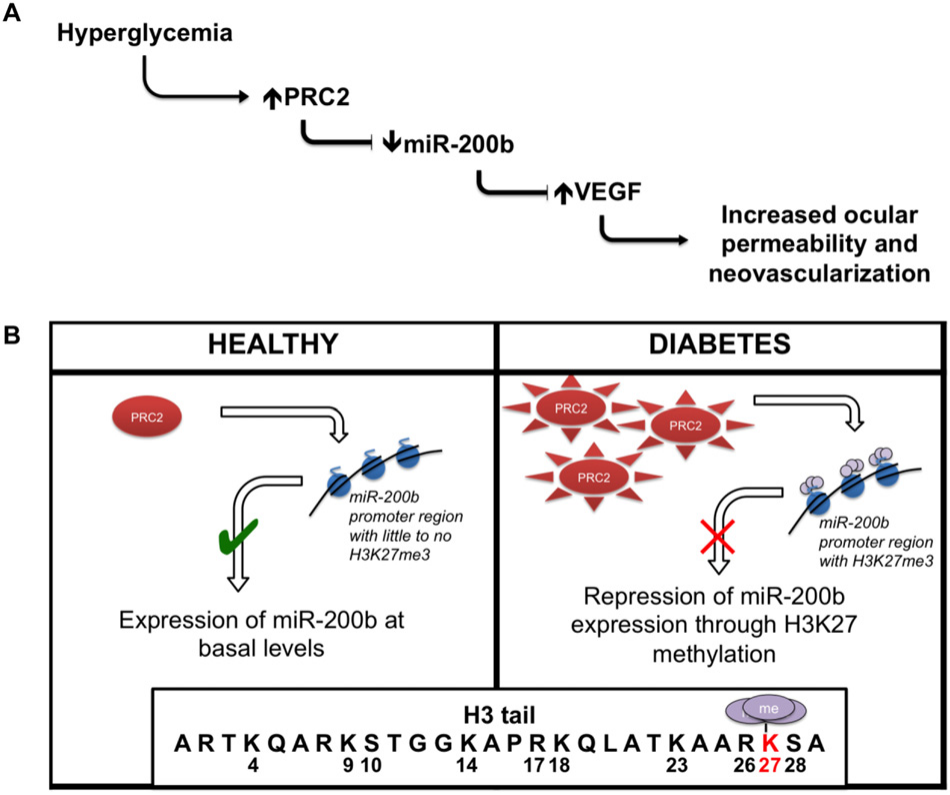
Summary of key findings. (A) Glucose-induced increased PRC2 expression and activity causes alteration of miR-200b levels. Such changes may play a role in the development of diabetic retinopathy. (B) This relationship is regulated at the level of genome by PRC2-mediated repression through H3K27me3 at the miR-200b promoter region.

In summary, we show that PRC2 regulates miR-200b in retinal endothelial cells through H3K27me3 repression (Fig 7). This is one of the first investigations to connect histone methylation and miRNA regulation in the context of diabetic retinopathy. Ultimately, this work builds on the characterization of miRNA our lab has studied by elucidating a new regulation mechanism. Investigating such mechanisms is important to further our understanding of the signaling events that occur in response to hyperglycemia in diabetic retinopathy, as well as developing novel treatment strategies to maintain the quality of life of patients with diabetes.

## References

[1] Davidson JA, Ciulla TA, McGill JB, Kles KA, Anderson PW. How the diabetic eye loses vision. Endocrine. 2007; 32: 107–116. 17992608 10.1007/s12020-007-0040-9

[2] Chistiakov DA. Diabetic retinopathy: pathogenic mechanisms and current treatments. Diabetes Metab Syndr. 2011; 5: 165–172. 10.1016/j.dsx.2012.02.025 22813573

[3] Chronopoulos A, Trudeau K, Roy S, Huang H, Vinores SA, Roy S. High glucose-induced altered basement membrane composition and structure increases trans-endothelial permeability: implications for diabetic retinopathy. Curr Eye Res. 2011; 36: 747–753. 10.3109/02713683.2011.585735 21780924

[4] American Diabetes Association. Implications of the United Kingdom Prospective Diabetes Study. Diabetes Care. 2002; 25: s28–s32. 10.2337/diacare.26.2007.s28 12502617

[5] Nathan DM, Cleary PA, Backlund JY, Genuth SM, Lachin JM, Orchard TJ, et al Intensive diabetes treatment and cardiovascular disease in patients with type 1 diabetes. N Engl J Med. 2005; 353: 2643–2653. 16371630 10.1056/NEJMoa052187 PMC2637991

[6] Aiello LP, DCCT/EDIC Research Group. Diabetic retinopathy and other ocular findings in the diabetes control and complications trial/epidemiology of diabetes interventions and complications study. Diabetes Care. 2014; 37: 17–23. 10.2337/dc13-2251 24356593 PMC3867989

[7] Mueckler M. Facilitative glucose transporters. Eur J Biochem. 1994; 219: 713–725. 8112322 10.1111/j.1432-1033.1994.tb18550.x

[8] Evans J, Goldfine ID, Maddux BA, Grodsky GM. Are oxidative stress activated signaling pathways mediators of insulin resistance and beta cell dysfunction? Diabetes. 2003; 52: 1–8. 12502486 10.2337/diabetes.52.1.1

[9] Zourek M, Kyselová P, Mudra J, Krcma M, Jankovec Z, Lacigová S, et al The relationship between glycemia, insulin and oxidative stress in hereditary hypertriglyceridemic rats. Physiol Res. 2008; 57: 531–538. 17705681 10.33549/physiolres.931255

[10] Yamagishi S. Advanced glycation end products and receptor-oxidative stress system in diabetic vascular complications. Ther Apher Dial. 2009; 13: 534–539. 10.1111/j.1744-9987.2009.00775.x 19954478

[11] Giacco F, Brownlee M. Oxidative stress and diabetic complications. Circ Res. 2010; 107: 1058–1070. 10.1161/CIRCRESAHA.110.223545 21030723 PMC2996922

[12] Son SM. Reactive oxygen and nitrogen species in pathogenesis of vascular complications of diabetes. Diabetes Metab J. 2012; 36: 190–198. 10.4093/dmj.2012.36.3.190 22737658 PMC3380122

[13] Tousoulis D, Briasoulis A, Papageorgiou N, Tsioufis C, Tsiamis E, Toutouzas K, et al Oxidative stress and endothelial function: therapeutic interventions. Recent Pat. Cardiovasc. Drug Discov. 2011; 6: 103–114. 21513492 10.2174/157489011795933819

[14] Huang A, Yang YM, Yan C, Kaley G, Hintze TH, Sun D. Altered MAPK signaling in progressive deterioration of endothelial function in diabetic mice. Diabetes. 2012; 1: 3181–3188. 10.2337/db12-0559 PMC3501862 22933112

[15] McArthur K, Feng B, Wu Y, Chen S, Chakrabarti S. MicroRNA-200b regulates vascular endothelial growth factor-mediated alterations in diabetic retinopathy. Diabetes. 2011; 60: 1314–1323. 10.2337/db10-1557 21357793 PMC3064105

[16] Feng B, Chen S, McArthur K, Wu Y, Sen S, Ding Q, et al miR-146a-Mediated extracellular matrix protein production in chronic diabetes complications. Diabetes. 2011; 60: 2975–2984. 10.2337/db11-0478 21885871 PMC3198068

[17] Lee Y, Kim M, Han J, Yeom KH, Lee S, Baek SH, et al MicroRNA genes are transcribed by RNA polymerase II. EMBO J. 2004; 23: 4051–4060. 15372072 10.1038/sj.emboj.7600385 PMC524334

[18] Ma E, Zhou K, Kidwell MA, Doudna JA. Coordinated activities of human dicer domains in regulatory RNA processing. J Mol Biol. 2012; 422: 466–476. 10.1016/j.jmb.2012.06.009 22727743 PMC3461841

[19] Bushati N, Cohen SM. MicroRNA functions. Annu Rev Cell Dev Biol. 2007; 23: 175–205. 17506695 10.1146/annurev.cellbio.23.090506.123406

[20] Ruiz MA, Chakrabarti S. MicroRNAs: the underlying mediators of pathogenetic processes in vascular complications of diabetes. Can J Diabetes. 2013; 37: 339–344. 10.1016/j.jcjd.2013.07.003 24500562

[21] Kumar M, Nath S, Prasad HK, Sharma GD, Li Y. MicroRNAs: a new ray of hope for diabetes mellitus. Protein Cell. 2012; 3: 726–738. 10.1007/s13238-012-2055-0 23055040 PMC4875345

[22] Schoof CR, Botelho EL, Izzotti A, Vasques Ldos R. MicroRNAs in cancer treatment and prognosis. Am J Cancer Res. 2012; 2: 414–433. 22860232 PMC3410578

[23] Cheng X, Blumenthal RM. Coordinated chromatin control: structural and functional linkage of DNA and histone methylation. Biochemistry. 2010; 49: 2999–3008. 10.1021/bi100213t 20210320 PMC2857722

[24] Villeneuve LM, Reddy MA, Lanting LL, Wang M, Meng L, Natarajan R. Epigenetic histone H3 lysine 9 methylation in metabolic memory and inflammatory phenotype of vascular smooth muscle cells in diabetes. Proc Natl Acad Sci USA. 2008 105: 9047–9052. 10.1073/pnas.0803623105 18579779 PMC2449362

[25] Brasacchio D, Okabe J, Tikellis C, Balcerczyk A, George P, Baker EK, et al Hyperglycemia induces a dynamic cooperativity of histone methylase and demethylase enzymes associated with gene-activating epigenetic marks that coexist on the lysine tail. Diabetes. 2009; 58:1229–1236. 10.2337/db08-1666 19208907 PMC2671038

[26] Syreeni A, El-Osta A, Forsblom C, Sandholm N, Parkkonen M, Tarnow L, et al Genetic examination of SETD7 and SUV39H1/H2 methyltransferases and the risk of diabetes complications in patients with type 1 diabetes. Diabetes. 2011; 60: 3073–3080. 10.2337/db11-0073 21896933 PMC3198095

[27] Okabe J, Orlowski C, Balcerczyk A, Tikellis C, Thomas MC, Cooper ME, et al Distinguishing hyperglycemic changes by Set7 in vascular endothelial cells. Circ Res. 2012; 110: 1067–1076. 10.1161/CIRCRESAHA.112.266171 22403242

[28] Di Croce L, Helin K. Transcriptional regulation by Polycomb group proteins. Nat Struct Mol Biol. 2013; 20: 1147–1155. 10.1038/nsmb.2669 24096405

[29] Tan JZ, Yan Y, Wang XX, Jiang Y, Xu HE. EZH2: biology, disease, and structure-based drug discovery. Acta Pharmacol Sin. 2014; 35: 161–174. 10.1038/aps.2013.161 24362326 PMC3914023

[30] Lu C, Han HD, Mangala LS, Ali-Fehmi R, Newton CS, Ozbun L, et al Regulation of tumor angiogenesis by EZH2. Cancer Cell. 2010; 18: 185–197. 10.1016/j.ccr.2010.06.016 20708159 PMC2923653

[31] Deb G, Thakur VS, Gupta S. Multifaceted role of EZH2 in breast and prostate tumorigenesis: epigenetics and beyond. Epigenetics. 2013; 8: 464–476. 10.4161/epi.24532 23644490 PMC3741216

[32] Chadwick BP, Willard HF. SETting the stage. Eed-Enx1 leaves an epigenetic signature on the inactive X chromosome. Dev Cell. 2003; 4: 445–447. 12689584 10.1016/s1534-5807(03)00097-2

[33] Cao R, Zhang Y. SUZ12 is required for both the histone methyltransferase activity and the silencing function of the EED-EZH2 complex. Mol Cell. 2004; 15: 57–67. 15225548 10.1016/j.molcel.2004.06.020

[34] Chang CJ, Hung MC. The role of EZH2 in tumour progression. Br J Cancer. 2012; 106: 243–247. 10.1038/bjc.2011.551 22187039 PMC3261672

[35] Dreger H, Ludwig A, Weller A, Stangl V, Baumann G, Meiners S, et al Epigenetic regulation of cell adhesion and communication by enhancer of zeste homolog 2 in human endothelial cells. Hypertension. 2012; 60: 1176–1183. 10.1161/HYPERTENSIONAHA.112.191098 22966008

[36] Au SL, Wong CC, Lee JM, Fan DN, Tsang FH, Ng IO, et al Enhancer of zeste homolog 2 epigenetically silences multiple tumor suppressor microRNAs to promote liver cancer metastasis. Hepatology. 2012; 56: 622–631. 10.1002/hep.25679 22370893

[37] Feng B, Wang R, Chen LB. Review of MiR-200b and cancer chemosensitivity. Biomed Pharmacother. 2012; 66: 397–402. 10.1016/j.biopha.2012.06.002 22795796

[38] Vrba L, Garbe JC, Stampfer MR, Futscher BW. Epigenetic regulation of normal human mammary cell type-specific miRNAs. Genome Res. 2011; 21: 2026–2037. 10.1101/gr.123935.111 21873453 PMC3227093

[39] Miranda TB, Cortez CC, Yoo CB, Liang G, Abe M, Kelly TK, et al DZNep is a global histone methylation inhibitor that reactivates developmental genes not silenced by DNA methylation. Mol Cancer Ther. 2009; 8: 1579–1588. 10.1158/1535-7163.MCT-09-0013 19509260 PMC3186068

[40] Rudney H. The utilization of L-glucose by mammalian tissues and bacteria. Science. 1940; 92: 112–113. 17755265 10.1126/science.92.2379.112

[41] Yan J, Zhang Z, Shi H. HIF-1 is involved in high glucose-induced paracellular permeability of brain endothelial cells. Cell Mol Life Sci. 2012; 69: 115–128. 10.1007/s00018-011-0731-5 21617913 PMC11115066

[42] Cao P, Deng Z, Wan M, Huang W, Cramer SD, Xu J, et al MicroRNA-101 negatively regulates Ezh2 and its expression is modulated by androgen receptor and HIF-1alpha/HIF-1beta. Mol Cancer. 2010; 17: 108. 10.1186/1476-4598-9-108 PMC2881117 20478051

[43] Bao B, Ahmad A, Kong D, Ali S, Azmi AS, Li Y, et al Hypoxia induced aggressiveness of prostate cancer cells is linked with deregulated expression of VEGF, IL-6 and miRNAs that are attenuated by CDF. PLOS ONE. 2012; 7: e43726 10.1371/journal.pone.0043726 22952749 PMC3428287

[44] Bao B, Ali S, Banerjee S, Wang Z, Logna F, Azmi AS, et al Curcumin analogue CDF inhibits pancreatic tumor growth by switching on suppressor microRNAs and attenuating EZH2 expression. Cancer Res. 2012; 72: 335–345. 10.1158/0008-5472.CAN-11-2182 22108826 PMC3792589

[45] Kotake Y, Nakagawa T, Kitagawa K, Suzuki S, Liu N, Kitagawa M, et al Long non-coding RNA ANRIL is required for the PRC2 recruitment to and silencing of p15(INK4B) tumor suppressor gene. Oncogene. 2011; 30: 1956–1962 10.1038/onc.2010.568 21151178 PMC3230933

[46] Kanhere A, Viiri K, Araújo CC, Rasaiyaah J, Bouwman RD, Whyte WA, et al Short RNAs are transcribed from repressed polycomb target genes and interact with polycombrepressive complex-2. Mol Cell. 2010; 38: 675–688. 10.1016/j.molcel.2010.03.019 20542000 PMC2886029

[47] Ho L, Crabtree GR. An EZ mark to miss. Cell Stem Cell. 2008; 3: 577–578. 10.1016/j.stem.2008.11.007 19041770 PMC2704610

[48] Cao R, Zhang Y. The functions of E(Z)/EZH2-mediated methylation of lysine 27 in histone H3. Curr Opin Genet Dev. 2004; 14: 155–164. 15196462 10.1016/j.gde.2004.02.001

